# Optimal primary wound closure methods after thyroid and parathyroid surgery: network meta-analysis of randomized clinical trials

**DOI:** 10.1093/bjsopen/zrac170

**Published:** 2023-02-23

**Authors:** Matthew G Davey, Ferdia Browne, Martin S Davey, Stewart R Walsh, Michael J Kerin, Aoife J Lowery

**Affiliations:** Discipline of Surgery, The Lambe Institute for Translational Research, National University of Ireland, Galway, Galway H91 YR71, Ireland; Discipline of Surgery, The Lambe Institute for Translational Research, National University of Ireland, Galway, Galway H91 YR71, Ireland; Discipline of Surgery, The Lambe Institute for Translational Research, National University of Ireland, Galway, Galway H91 YR71, Ireland; Discipline of Surgery, The Lambe Institute for Translational Research, National University of Ireland, Galway, Galway H91 YR71, Ireland; Discipline of Surgery, The Lambe Institute for Translational Research, National University of Ireland, Galway, Galway H91 YR71, Ireland; Discipline of Surgery, The Lambe Institute for Translational Research, National University of Ireland, Galway, Galway H91 YR71, Ireland

## Abstract

**Background:**

At present, there is no consensus on optimal neck wound closure methods after thyroid and parathyroid surgery. The aim of this study was to perform a systematic review and network meta-analysis of RCTs evaluating the optimal neck closure method after thyroid and parathyroid surgery.

**Methods:**

A frequentist random-effects network meta-analysis was performed for RCTs comparing at least two closure methods according to PRISMA-network meta-analysis guidelines. Analysis was performed using R packages and Shiny.

**Results:**

Eighteen RCTs evaluating six closure methods (that is adhesive (28.5 per cent, 404 patients), absorbable subcuticular suture (18.1 per cent, 257 patients), non-absorbable subcuticular suture (16.8 per cent, 238 patients), staples (26.3 per cent, 372 patients), steristrips (8.1 per cent, 115 patients), and conventional suture (2.1 per cent, 30 patients)) in 1416 patients were included. At network meta-analysis, there was no difference in complication, infection, dehiscence, or haematoma rates irrespective of closure method used. Staples reduced closure duration *versus* absorbable subcuticular suture (mean difference (MD) 8.50, 95 per cent c.i. 6.90 to 10.10) and non-absorbable subcuticular suture (MD 0.30, 95 per cent c.i. 0.23 to 0.37), whereas adhesives (MD −1.05, 95 per cent c.i. −1.31 to −0.79) reduced closure time relative to staples. Cosmesis was improved after non-absorbable subcuticular suture (odds ratio (OR) 3.41, 95 per cent c.i. 1.66 to 7.00) relative to staples. Staples reduced patient satisfaction (OR 0.04, 95 per cent c.i. 0.00 to 0.33) and ability to shower (OR 0.04, 95 per cent c.i. 0.00 to 0.33) relative to adhesives.

**Conclusion:**

Despite staples decreasing closure times, this advantage is offset by reduced patient satisfaction, ability to shower, and cosmesis compared with patients with wounds closed using adhesives, absorbable subcuticular suture, and non-absorbable subcuticular suture. Therefore, these closure methods are favourable for closing neck wounds due to more acceptable patient-reported outcomes, without compromising the safety of the procedure.

## Teaser

This is a systematic review and network meta-analysis evaluating the utility of varying wound closure methods after thyroid and parathyroid surgery. The results illustrate that subcuticular sutures are favourable for closing neck wounds due to more acceptable patient-reported outcomes compared with other closure methods, without compromising safe closure.

## Introduction

Thyroid and parathyroid surgeries are increasingly common in the Western world, with a combined 120 000 of these surgeries being performed in the USA each year, including both benign and malignant disease indications^1–3^. The surgical approach to these endocrine glands typically involves an anterior cervical (or ‘Kocher’) incision, which is located in a highly visible (as typically not covered by clothing) and potentially sensitive area based on its anatomical location on the anterior aspect of the patient’s neck. In recent years, remote-access approaches (that is transoral, trans-axillary, and axillo-breast approaches) to resecting these glands have been pioneered with the purpose of improving cosmesis, increasing patient satisfaction, and reducing postoperative complications^4,5^. Despite these novel developments, most surgeons still rely upon using the conventional open anterior cervical incisions when performing thyroid or parathyroid surgery for the majority of cases^6,7^.

At present, there is no consensus in relation to the optimal closing method after thyroid and parathyroid surgery. There are several approaches: adhesives, which involve closing the wound using monomer liquid glues that polymerize at contact with tissue anions, forming a strong bond that holds wound edges together (favourable qualities of adhesives include flexibility and strength, as well as the absence of requiring sharps for skin closure)^8^; subcuticular sutures, which are commonly intradermal stitches placed immediately below and bring tension to the epidermal layer (subcuticular sutures are thought to be advantageous as they cause less tissue reaction and absence of marks on the skin and may be absorbable or non-absorbable)^9^; staples (or metal clips), which are applied to the external epidermis and bring the dermal edges of the wound together (staples typically require less closure time and have been reported to carry lower infection rates, but result in increased postoperative pain, compared with other closure methods)^10,11^; steristrips, which are adhesive skin closure bands or strips used to bring opposing edges of a wound together^12^; and conventional interrupted (or ‘loop’) sutures (CS), which are renowned for being time-consuming and cosmesis results being operator dependent^13–15^. As previously outlined, there is no consensus regarding wound closure methods after thyroid and parathyroid surgery and selection is left at the discretion of the surgeon.

Several parameters are to be considered for neck wound closures. While cosmesis and pain have become significantly more important for patients in recent years, traditional postoperative wound complications, such as surgical site infections, wound dehiscence, and haematoma rates, remain very important to the surgeon and the patient^16,17^. The incidence of neck haematomas after thyroid and parathyroid surgery is rare^18,19^, but these events are potentially life-threatening due to impending airway obstruction^20^. However, postoperative neck haematoma formation is typically due to inadequate haemostasis within the surgical field^21^, rather than ineffective skin closure. Nevertheless, wound closure methods are of the utmost importance when optimizing both surgical and patient outcomes.

While there have been previous systematic reviews and meta-analyses performed for determining the optimal wound closure^22,23^, there are several shortcomings to such studies: failure to include all available RCT data to inform outcomes;^22^ and failure to implement a network meta-analysis (NMA) methodology to ensure robust evaluation of all potential closure methods. The advantage of an NMA is the simultaneous comparison of more than two treatments with a control or standard-of-care option^24,25^. Therefore, the aim of the present study was to perform a systematic review and NMA of RCTs evaluating the optimal neck closure after thyroid and parathyroid surgery.

## Methods

A systematic review was performed in accordance with the PRISMA extension statement for reporting of systematic reviews incorporating NMAs of healthcare interventions^26^. Local institutional ethical approval was not required as all data used in this analysis were obtained from previously published studies. Each author contributed to formulating the study protocol. This study was prospectively registered with the International Prospective Register of Systematic Reviews (PROSPERO—CRD42021284857).

### Study eligibility

All published RCTs with full-text manuscripts comparing outcomes of at least two methods of closing neck wounds after thyroid or parathyroid surgery were included. Studies were not excluded based on patient demographics, histopathological tumour features, or surgical parameters. Studies reporting outcomes of surgeries for both cancerous and non-cancerous pathologies were included. Only studies that reported data relating to the predefined outcomes of interest were included in this systematic review. Studies comparing closure methods for other neck surgeries (for example laryngectomy, branchial cleft cyst removal, anterior cervical discectomy and fusion, etc.) were excluded, as well as studies comparing outcomes after lateral neck dissections only. Included RCTs had to provide full-text manuscripts, had to be published in the English language, and were not restricted by year of publication.

### Population, intervention, comparison, outcomes

Using the population, intervention, comparison, outcomes (PICO) framework^27^, the following aspects were addressed:

Population—All patients who underwent thyroid or parathyroid surgery.

Intervention—Any patient who underwent neck wound closure after thyroid or parathyroid surgery using any primary closure method.

Comparison—Any patient who underwent neck wound closure after thyroid or parathyroid surgery using other primary closure methods.

Outcomes—The outcomes of interest included:

- Overall postoperative complication rates.
- Postoperative wound infections.
- Rates of wound dehiscence.
- Postoperative neck erythema.
- Postoperative neck haematoma rates.
- Closure duration, defined as the time taken in minutes to close the skin.
- Patient-reported satisfaction with the wound, expressed as a dichotomous variable (that is satisfied or unsatisfied).
- Patient-reported ability to shower after operation, expressed as a dichotomous variable (that is able to or not able to shower).
- Patient-reported satisfaction with the cosmesis of the wound.
- Patient-reported postoperative pain, expressed using visual/verbal analogue scales (VAS)^28^.
- Patient-reported postoperative neck mobility.

### Search strategy

A formal systematic search of the PubMed, Embase and Cochrane Central Register of Controlled Trials (CENTRAL) electronic databases was performed for relevant titles. This search was performed by two independent reviewers (M.G.D. and F.B.), using a predetermined search strategy that was designed by the senior authors. Two searches of these databases were performed. The first search was performed using the following search terms: (Neck surgery), (Closure). Thereafter, the second search was performed using the following search terms: (Thyroid), (Parathyroid), (Closure). Both searches were linked using the Boolean operators ‘AND’ and ‘OR’. Manual cross-referencing of reference lists from previous systematic reviews, meta-analyses, and included trials was undertaken to ensure that all RCTs published with full-text manuscripts were captured in this study.

Manual removal of duplicate studies was performed before all titles were screened. Thereafter, RCTs considered to be appropriate had their abstracts and/or full text reviewed. Retrieved studies were reviewed to ensure inclusion criteria were met for the outcome measures listed, with discordances in opinion resolved through consultation with the senior author (A.J.L.). Data extraction was also performed by two independent reviewers (M.G.D. and F.B.), with study details, basic patient clinicopathological characteristics, and surgical data all recorded. The final search was performed in November 2022.

### Data management and analysis

Descriptive statistics were used to outline characteristics of included trials. Rates of postoperative complication, haematoma, infection, dehiscence, erythema, satisfaction, and ability to shower were expressed as dichotomous or binary outcomes, and reported as odds ratios (ORs) expressed with 95 per cent confidence intervals (c.i.). ORs were calculated, using crude-event RCT data, to compare interventions using per-protocol data, where applicable. Postoperative pain, overall cosmesis, and closure duration were reported as mean weighted difference, calculated from mean values and associated standard deviations (s.d.). The principal comparator varied between each analysis depending on the closure methods included.

Bayesian NMAs were conducted using Netameta and Shiny packages for R^29^. Effect sizes were described with a 95 per cent c.i. Results were considered statistically significant at the *P* < 0.050 level if the 95 per cent c.i. did not include the value of 1. Estimates of mean and s.d. were calculated using standard statistical methods, where applicable^30,31^. Rank probabilities were plotted against the possible ranks for all competing treatments. The confidence in estimates of the outcome was assessed using ‘Confidence In Network Meta-Analysis’ (CINeMA)^32^. Methodological assessment of included studies was undertaken using the Cochrane risk-of-bias assessment tool, as outlined by Higgins *et al*.^33^. For example, if included studies clearly reported their randomization using unbiased methodology (for example computer-generated randomization, sealed envelope, etc.), then the random sequence generation bias was considered to be ‘low’. If details regarding patient randomization were ambiguous or not reported, the risk of bias of the study was considered ‘unclear’, and if there was no obvious randomization, studies were considered to be at ‘high’ risk of bias.

## Results

### Literature search and study characteristics

**Fig. 1:**
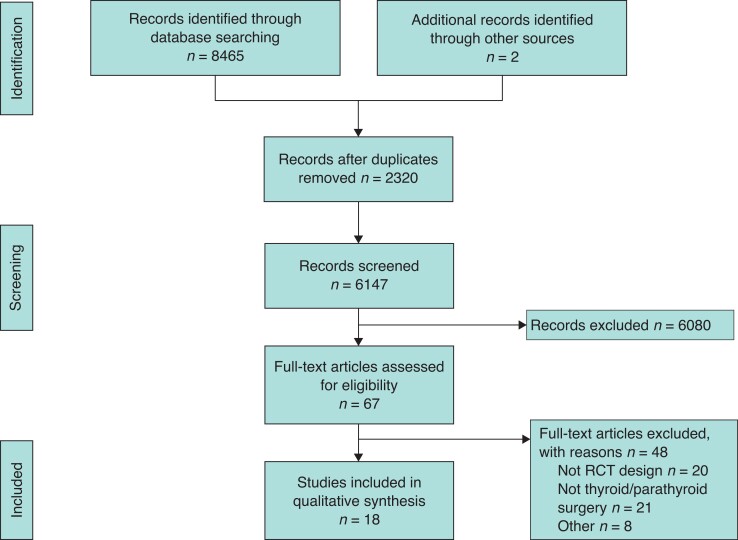
PRISMA flow chart illustrating the systematic search process

The systematic search strategy identified a total of 8467 studies, of which 2320 duplicate studies were manually removed. The remaining 6147 studies were screened for relevance, before 67 full texts were reviewed. In total, 18 RCTs fulfilled the inclusion criteria and were included in this systematic review (*Fig. 1*)^34–51^. In these studies, there were data from 1416 patients and six closure methods (that is adhesive (28.5 per cent, 404 patients, 10 RCTs)^35–38,40,42,44,45,49,50^, absorbable subcuticular suture (ASCS) (18.1 per cent, 257 patients, 7 RCTs)^35,37,39,41,44,47,50^, non-absorbable subcuticular suture (NASCS) (16.8 per cent, 238 patients, 6 RCTs)^34,43,46,48,49,51^, staples (26.3 per cent, 372 patients, 11 RCTs)^34,36,38,40–43,45–47,51^, steristrips (8.1 per cent, 115 patients, 3 RCTs)^39,46,48^, and CS (2.1 per cent, 30 patients, 1 RCTs)^51^. Of the 18 RCTs included in this analysis, 44.4 per cent were conducted in European surgical research institutions (8/18)^35–37,39–43^. Publication dates ranged from 1997 to 2021 (*Table 1*).

**Table 1:** Data from the 18 RCTs included in this study

Author Year	Country	Total	Adhesive	ASCS	Staples	NASCS	Steristrips	CS
Alicandri-Ciufelli 2014	Italy	89	42	47	–	–	–	–
Amin 2009	Ireland	60	33	–	27	–	–	–
Chung 2021	Korea	126	84	42	–	–	–	–
Consorti 2013	Italy	50	25	25	–	–	–	–
Iqbal 2014	Pakistan	100	–	–	48	52	–	–
Jayaram 2021	India	93	–	–	31	31	31	–
Ku 2020	Korea	37	–	19	18	–	–	–
Maw 1997	Canada	50	24	–	26	–	–	–
O'Leary 2014	Ireland	82	–	43	–	–	39	–
Pronio 2011	Italy	70	32	–	38	–	–	–
Rani Challa 2020	India	90	–	–	30	30	–	30
Vinay R 2021	India	74	36	–	–	38	–	–
Reed 1997	UK	68	–	34	34	–	–	–
Ridgway 2009	UK	29	14	–	15	–	–	–
Selvadurai 1997	UK	80	–	–	38	42	–	–
Teoh 2019	Malaysia	96	49	47	–	–	–	–
Vinay G 2017	India	90	–	–	45	45	45	–
Yang 2013	China	132	65	–	67	–	–	–
Total		1416	404	257	372	238	115	30

Values are *n*. ASCS, absorbable subcuticular suture; NASCS, non-absorbable subcuticular suture; CS, conventional suture.

### Clinicopathological and surgical characteristics

In this systematic review, there were data included from 1416 patients with a mean age at diagnosis of 49.2 years (range: 17–76). In total, 78.2 per cent of patients were female (1084/1387) and most patients underwent neck surgery for non-cancerous pathologies (88.1 per cent, 726/824). Of the 939 patients with surgical data reported, 42.4 per cent underwent total thyroidectomy (398/939), 26.2 per cent underwent hemi-thyroidectomy (246/939), 17.8 per cent underwent subtotal thyroidectomy (167/939), and 13.6 per cent underwent parathyroidectomy (128/939). The mean incision length was 6.6 cm (range: 2.0–11.0) and the mean operation duration was 82.3 min. The breakdown of clinicopathological and surgical data from the 18 included RCTs is outlined in *Table S1*.

### Overall complication rates

**Fig. 2:**
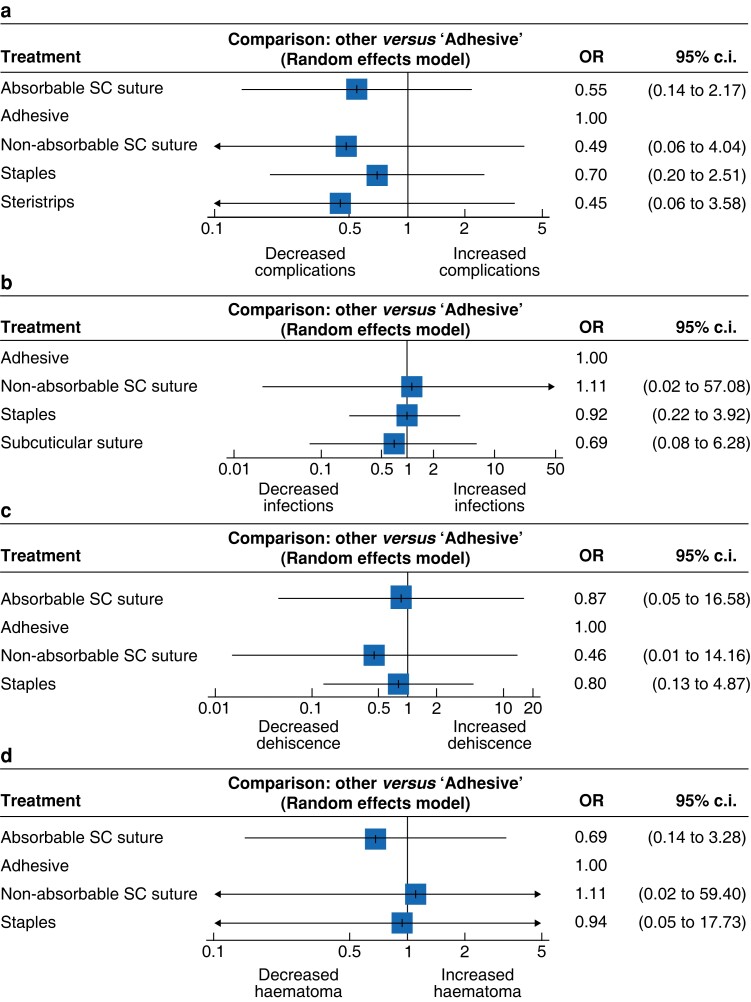
Forest plots comparing adhesives with other wound closure methods **a** Overall complications. **b** Infection. **c** Dehiscence. **d** Haematoma. SC, subcuticular.

Ten RCTs reported outcomes for overall complication rates after thyroid and parathyroid surgery^35–39,42,43,45,47,49^. The reported overall complication rate was 3.8 per cent (26/683) (*Table 2*). The overall complication rates after use of steristrips, adhesive, ASCS, staples, and NASCS for wound closure were 7.7 per cent (3/39), 5.4 per cent (13/239), 5.2 per cent (7/134), 1.6 per cent (3/191), and 0.0 per cent (0/80) respectively. At NMA, there was no significant difference in overall complication rates based on closure method (*Fig. 2a*). Individual study results and the network plots for overall complication rates are outlined in *Fig. S1a*.

**Table 2:** Surgical complication data for all methods of wound closure in the included RCTs

Intervention (number of RCTs)	Patients	Complications	Infection	Dehiscence	Erythema	Haematoma
Adhesive (10)	404 (28.5)	13/239 (5.4)	3/221 (1.4)	1/169 (0.6)	0/33 (0.0)	6/100 (6.0)
ASCS (7)	257 (18.1)	7/134 (5.2)	0/91 (0.0)	0/44 (0.0)	0/19 (0.0)	3/91 (3.3)
Staples (11)	372 (26.3)	3/191 (1.6)	2/214 (0.9)	0/150 (0.0)	0/45 (0.0)	0/83 (0.0)
NASCS (6)	238 (16.8)	0/80 (0.0)	0/42 (0.0)	0/38 (0.0)	–	0/42 (0.0)
Steristrips (3)	115 (8.1)	3/39 (7.7)	–	–	–	–
CS (1)	30 (2.1)	–	–	–	–	–
Total	1416 (100.0)	26/683 (3.8)	5/568 (0.9)	1/401 (0.3)	0/97 (0.0)	9/316 (2.9)

Values are *n* (%) or *n*/*n* (%). ASCS, absorbable subcuticular suture; NASCS, non-absorbable subcuticular suture; CS, conventional suture.

### Postoperative infection rates

In total, eight RCTs reported outcomes relating to postoperative surgical site infections^35–38,40,43,45,47^. The overall reported postoperative infection rate was 0.9 per cent (5/568) (*Table 2*). Infection rates using adhesive, staples, ASCS, and NASCS to close neck wounds were 1.4 per cent (3/221), 0.9 per cent (2/214), 0.0 per cent (0/91), and 0.0 per cent (0/42) respectively. At NMA, there was no significant difference in infection rates observed for these primary closure methods (*Fig. 2b*). Individual study results and the network plots for these studies assessing postoperative infection rates are outlined in *Fig. S1b*.

### Wound dehiscence rates

Seven RCTs reported outcomes regarding wound dehiscence after thyroid and parathyroid surgery^36,37,40,42,45,47,49^. The overall wound dehiscence rate was 0.3 per cent (1/401) (*Table 2*). Wound dehiscence rates while using adhesive, staples, ASCS, and NASCS to close neck wounds were 0.6 per cent (1/169), 0.0 per cent (0/150), 0.0 per cent (0/44), and 0.0 per cent (0/38) respectively. At NMA, there was no significant difference in wound dehiscence rates observed for these primary closure methods (*Fig. 2c*). Individual study results and the network plots for these studies assessing wound dehiscence rates are outlined in *Fig. S1c*.

### Erythema

Two RCTs reported outcomes pertaining to postoperative erythema after thyroid and parathyroid surgery^36,47^. No patients had erythema (0.0 per cent, 0/97). None of the patients undergoing adhesive, staple, or ASCS closure of their neck wounds reported postoperative erythema (0/33, 0/45, and 0/19 respectively) (*Table 2*).

### Haematoma rates

Five RCTs reported outcomes regarding haematoma rates after thyroid and parathyroid surgery^35–37,43,47^. The overall reported haematoma rate was 2.9 per cent (9/316) (*Table 2*). The haematoma rates after use of adhesive, ASCS, staples, and NASCS to close neck wounds were 6.0 per cent, (6/100), 3.3 per cent (3/91), 0.0 per cent (0/83), and 0.0 per cent (0/42) respectively. At NMA, there was no significant difference in haematoma rates observed for these primary closure methods (*Fig. 2d*). Individual study results and the network plots for these studies assessing haematoma rates are outlined in *Fig. S1d*.

### Closure duration

**Fig. 3:**
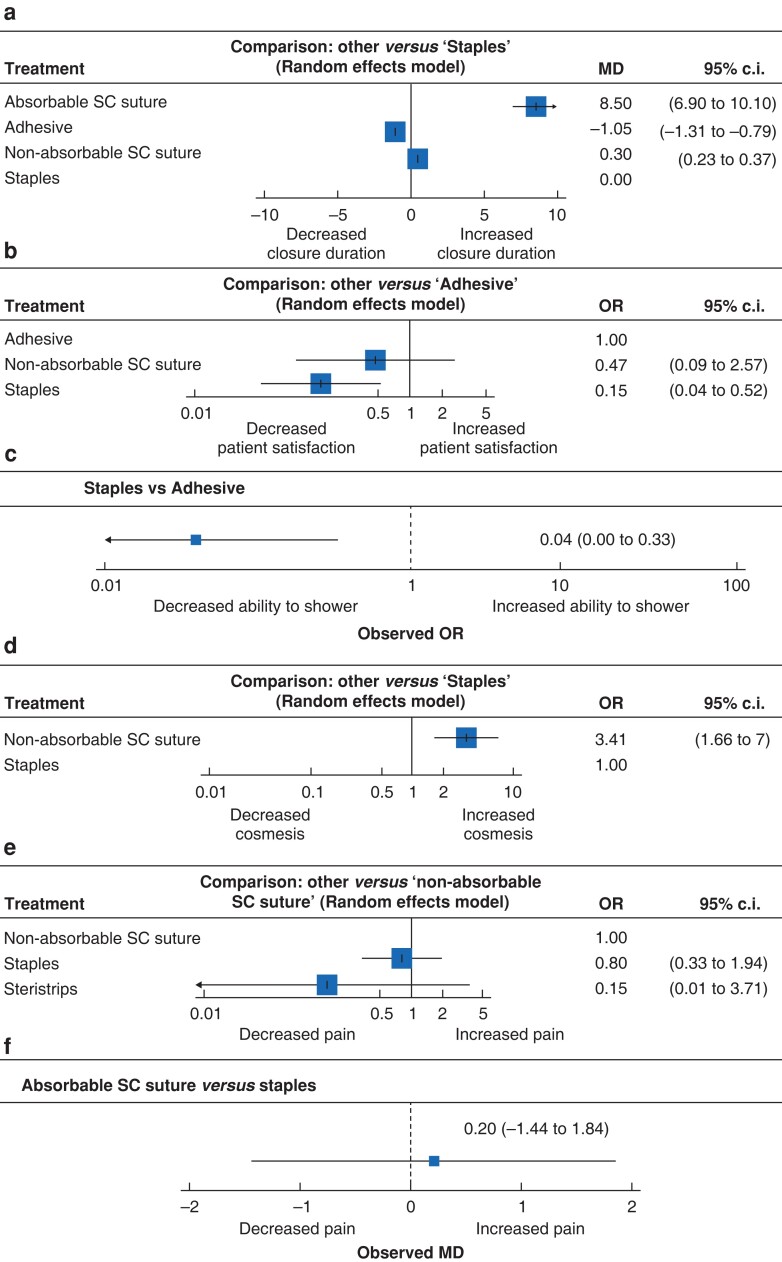
Forest plots comparing wound closure methods **a** Closure duration. **b** Patient satisfaction. **c** Ability to shower. **d** Patient-reported cosmesis. **e** Postoperative pain expressed as odds ratios. **f** Postoperative pain expressed as mean difference. SC, subcuticular; MD, mean difference.

Four RCTs reported outcomes regarding the intraoperative time taken for neck closure^38,42,43,47^. The mean time taken to close neck wounds was 4.00 min (mean s.d. 0.96). The mean time taken to close wounds using ASCS, NASCS, staples, and adhesive was 12.00, 4.50, 3.66, and 0.5 min respectively. At NMA, ASCS (mean difference (MD) 8.50, 95 per cent c.i. 6.90 to 10.10) and NASCS (MD 0.30, 95 per cent c.i. 0.23 to 0.37) increased closure time relative to staples, whereas adhesives (MD −1.05, 95 per cent c.i. −1.31 to −0.79) reduced closure time relative to staples (*Fig. 3a*). Individual study results and the network plots for these studies assessing closure duration are outlined in *Fig. S1e*.

### Overall patient satisfaction

Three RCTs reported patient satisfaction rates with their neck closure after thyroid and parathyroid surgery^34,36,40^. The overall patient satisfaction rate was 78.7 per cent (181/230) (*Table 3*). Overall, 93.9 per cent, 86.5 per cent, and 73.5 per cent of patients who had their neck wounds closed using adhesive (61/65), NASCS (45/52), and staples (75/102) were satisfied with their wound closure respectively. At NMA, staples were associated with reduced patient satisfaction (OR 0.04, 95 per cent c.i. 0.00 to 0.33) compared with adhesives (*Fig. 3b*). Individual study results and the network plots for these studies assessing patient satisfaction rates are outlined in *Fig. S1f*.

**Table 3:** Patient-reported outcomes for all methods of wound closure in the included RCTs

Intervention (number of RCTs)	Patients	Satisfaction	Ability to shower	Satisfied with cosmesis	Free of pain after operation
Adhesive (10)	404 (28.5)	61/65 (93.9)	95/98 (97.0)	–	–
ASCS (7)	257 (18.1)	–	–	–	45/45 (100.0)
Staples (11)	372 (26.3)	75/113 (66.4)	38/94 (40.4)	54/88 (61.4)	16/38 (42.1)
NASCS (6)	238 (16.8)	45/52 (86.5)	–	80/94 (85.1)	20/42 (47.6)
Steristrips (3)	115 (8.1)	–	–	–	43/45 (95.6)
CS (1)	30 (2.1)	–	–	–	–
Total	1416 (100.0)	181/230 (78.7)	133/912 (69.3)	134/182 (73.6)	124/170 (73.0)

Values are *n* (%) or *n*/*n* (%). ASCS, absorbable subcuticular suture; NASCS, non-absorbable subcuticular suture; CS, conventional suture.

### Ability to shower

Two RCTs reported patient ability to shower after thyroid and parathyroid surgery^36,45^. The overall postoperative ability to shower rate was 69.3 per cent (133/192) (*Table 3*). Overall, 97.0 per cent and 40.4 per cent of patients who had their neck wound closed using adhesive and staples reported the ability to shower after surgery (95/98 *versus* 38/94) respectively. At NMA, staples significantly reduced patient ability to shower (OR 0.04, 95 per cent c.i. 0.00 to 0.33) relative to adhesives (*Fig. 3c*). Network plots for these studies assessing patient satisfaction rates are outlined in *Fig. S1g*.

### Cosmesis

Fourteen RCTs described patient-reported cosmesis after thyroid and parathyroid surgery^34–40,43–45,47,49,50^. Overall, 73.6 per cent of patients were satisfied with their cosmesis after operation (134/182); 85.1 per cent (80/94) and 61.4 per cent (54/88) of those who had NASCS and staples for wound closure respectively were satisfied with the cosmetic outcome of their wound (*Table 3*). At NMA, using subcuticular sutures (OR 3.41, 95 per cent c.i. 1.66 to 7.00) improved patient-reported cosmesis relative to staples (*Fig. 3d*). Network plots for these studies assessing patient-reported cosmesis are outlined in *Fig. S1h*. All the cosmesis scales are outlined in *Table S2*.

### Postoperative pain

Ten RCTs reported postoperative pain^36,38,42,43,45,47–51^. Of these, two studies reported postoperative pain as a dichotomous outcome. Overall, 73.0 per cent of patients were free of postoperative pain (124/170). Of these, 100.0 per cent (45/45), 95.6 per cent (43/45), 47.6 per cent (20/42), and 42.1 per cent (16/38) of patients were free of postoperative pain after use of ASCS, steristrips, NASCS, and staples to close neck wounds respectively (*Table 3*). At NMA, there was no significant difference in postoperative pain observed for these primary closure methods (*Fig. 3e,f*). Overall, the reported mean(s.d.) VAS was 6.8(3.4); the mean VAS for staples and ASCS was 6.9 and 6.7 respectively. Network plots for these studies assessing patient satisfaction rates are outlined in *Fig. S1i,j*. Included studies reporting postoperative pain outcomes using pain rating scales are outlined in *Table S2*.

### Neck mobility

Two RCTs reported patient outcomes in relation to neck mobility after thyroid and parathyroid surgery^42,48^. The mean(s.d.) VAS for neck mobility was 6.2(3.2). Patients who had their neck closed with a subcuticular suture had better neck mobility compared with those who had their neck wound closed using steristrips (mean(s.d.) VAS 7.3(2.2) *versus* 5.6(4.3)). Included studies reporting postoperative neck mobility using rating scales are outlined in *Table S2*.

### Risk-of-bias assessment

There was a low-to-moderate risk of bias among the included RCTs. Overall, 3 of the RCTs had a low risk of bias (18.8 per cent)^36,39,44^, 13 of the RCTs included some concerns for bias (75.0 per cent)^34,35,37,38,40,42,43,45–50^, and 2 RCTs were considered to have a high risk of bias (6.2 per cent)^41,51^. Risk-of-bias assessment is illustrated in *Table S3*.

## Discussion

The most important finding in this study is the data highlighting the inferiority of using staples compared with ASCS, NASCS, or adhesives regarding patient-reported outcomes when performing skin closures after thyroid and parathyroid surgery. While this analysis revealed no differences in complication, infection, dehiscence, and haematoma rates at NMA irrespective of closure method, these results are offset by the poorer patient-reported outcomes (that is patient satisfaction, cosmesis, and inability to shower) after stapled wound closures. Therefore, while this analysis does not identify a single neck wound closure method as optimal in terms of both surgical and patient-reported outcomes, it certainly illustrates the negative impact of using staple closures on patient-reported outcomes in the postoperative setting. Therefore, the use of ASCS, NASCS, or adhesives for neck closures in ‘real-world’ surgical practice is preferable.

In this NMA, the authors observed no difference in overall complication, surgical site infections, wound dehiscence, and haematoma rates irrespective of wound closure method used. As these results fall short of informing optimal wound closure methods, emphasis should be placed upon the patient-reported outcomes and surgical practicalities of each closure method. Patients reported significantly lower satisfaction and less ability to shower with staples compared with the use of adhesives, as well as increased cosmesis after NASCS compared with stapled closures. Notwithstanding these promising patient-reported outcomes for other closure methods, staples were associated with a significant decrease in operative time compared with ASCS, NASCS, and adhesives. Traditionally, surgical staples were pragmatically used to ensure swift removal in the event of an expanding postoperative neck haematoma. Staples are advantageous as they may be quickly applied and removed if necessary^52^, which is illustrated coherently in the results of this NMA. Albeit important, the other data in this study negate the historical use of skin staples for reduced complications after a neck haematoma, thus refuting their use as a routine requirement, as this benefit is likely offset by the inferior patient-reported outcomes associated with staples as a closure method after neck surgery. Furthermore, the British Association of Endocrine and Thyroid Surgeons provides guidelines for rapid opening of neck wounds in the event of an expanding neck haematoma, which are surgeon specific and tailored to each of the closure methods commonly utilized by surgeons, including subcuticular sutures^53^. While this analysis illustrates that there is no difference in surgical outcomes irrespective of what closure method is used, the results certainly highlight the superiority of ASCS, NASCS, and adhesives for improving the experience of patients in the postoperative setting.

As described, patients reported lower satisfaction, poorer cosmesis, and lower ability to shower after stapled closure when compared with adhesives, steristrips, and subcuticular sutures. Traditionally, surgical outcomes were considered the most important when gauging the success of surgery; however, the paradigm has evolved to recognize the significance of patient-reported outcomes as metrics of surgical success^54^. Measurement of patient-reported outcomes is important for surgeons to appreciate the subjective implications of surgery on a patient’s quality of life^55^, such as basic activities of daily living like showering. Interestingly, it is well reported that patients should be able to shower safely 24 h after neck surgery (and that showering does not increase wound infections)^56,57^, yet this study highlights apprehension among patients regarding showering, particularly after stapled closures. Therefore, this further weakens the routine use of surgical staples for neck wounds after thyroid and parathyroid surgery given the considerable inferiority in patient-reported outcomes. In particular, cosmesis is of utmost importance in the current management paradigm^17,58^.

This is not the first systematic review or meta-analysis performed to address the optimal wound closure method after thyroid and parathyroid surgery. Lee *et al*.^22^ previously performed a systematic review of cosmetic outcomes for 10 studies in 712 patients, illustrating the enhanced cosmetic outcomes achieved when using steristrips or subcuticular sutures after thyroid and parathyroid surgery. Huang *et al*.^23^ performed a meta-analysis of nine RCTs, which included 612 patients, and illustrated the cosmetic advantage associated with using subcutaneous sutures to improve cosmesis *versus* staples. While these analyses provide some clarity with respect to cosmetic outcomes after thyroid and parathyroid neck closures, the present study reports the combined surgical and patient-reported outcomes of 18 RCTs through the adoption of NMA methodology of six different primary closure methods after neck surgery. Therefore, the results of this NMA provide robust and novel data with clinical relevance.

This study is subject to limitations. First, the number of trials reporting available data for patient-reported outcomes, such as ability to shower and neck mobility, limited the extent of this NMA. Furthermore, patient-reported cosmesis was reported in 14 RCTs, yet just two provided sufficient data for integration at meta-analysis, because several different cosmesis scales were used in the included studies. Despite this, available data from the relevant trials were integrated and an analysis was performed to represent the cosmetic implications of such closure methods on patients. Second, as is common for RCTs reporting surgical outcomes, none of the RCTs included in this analysis was ‘blinded’. The inability to blind surgeons and patients to interventions makes these RTCs subject to unintentional bias^59^. The authors acknowledge this unavoidable limitation. Finally, this study fails to consider certain potential confounders (that is patient age, body mass indices, smoking habits, indications for surgery, etc.), which may impact the outcomes. Unfortunately, the authors were unable to perform subgroup analyses with respect to surgical procedures (that is thyroid *versus* parathyroid surgery) and pathology (that is cancerous *versus* non-cancerous) due to the lack of data from the included RCTs. This inevitably limits the results of this study.

## Supplementary Material

zrac170_Supplementary_Data

## Data Availability

Data made available upon reasonable request to the corresponding author.
