# Supplementary material for: Optimal primary wound closure methods after thyroid and parathyroid surgery: network meta-analysis of randomized clinical trials
Source: BJS Open. 2023 Feb 23;7(1):zrac170. doi: 10.1093/bjsopen/zrac170 (PMC9949711; doi:10.1093/bjsopen/zrac170)
Supplement: zrac170_Supplementary_Data [file zrac170_supplementary_data.zip › Supplementary_material.docx]

**Optimal Primary Wound Closure Methods following Thyroid and Parathyroid Surgery: Network Meta-Analysis of Randomized Clinical Trials**

Matthew G. Davey MCh MRCSI, Ferdia Browne MB BCh, Martin S. Davey MCh MRCSI,

Stewart R. Walsh MCh FRCSI, Michael J. Kerin MCh, FCRSI, Aoife J. Lowery PhD FRCSI

Discipline of Surgery, The Lambe Institute for Translational Research, National University of Ireland, Galway, Galway, Ireland H91YR71

**Corresponding author**: Dr. Matthew G. Davey, MCh MRCSI
**Correspondence to**: Department of Surgery, The Lambe Institute for Translational Research, National University of Ireland, Galway, Galway H91 YR71, Republic of Ireland.

**ORCID ID**: 0000-0002-9892-9920

**Twiiter**: mattdavey93

| **Supplementary Tables and Figures** |  |
| --- | --- |
| **Table S1.** Clinicopathological and surgical data from the 18 included randomised clinical trials. | *pag. 3* |
| **Figure S1.** Study data summary and network plots of all included individual studies assessing rates of (A), overall complications, (B) infection, (C), dehiscence, (D) haematoma, (E) closure duration, (F) patient satisfaction, (G) ability to shower, (H) patient reported cosmesis, and post-operative pain expressed as (I) odds ratio and (J) mean difference. | *pag. 4-7* |
| **Table S2.** Included randomised clinical trials and the cosmetic, pain, neck mobility scales used and risk of bias assessment for each trial | *Pag. 8* |
| **Table S3.** Detailed risk of bias assessment for each included randomised clinical trial. | *Pag. 9* |

**Table S1.** Clinicopathological and surgical data from the 18 included randomised clinical trials.

| Author | Year | Age | Female | Male | Incision (cm) | TT | STT | HT | PT | Duration | Benign | Cancer |
| --- | --- | --- | --- | --- | --- | --- | --- | --- | --- | --- | --- | --- |
| Alicandri-Ciufelli | 2014 | 53.1 | 69 | 20 | 6.9 (4-11) | - | - | - | - | - | - | - |
| Amin | 2009 | 48.9 (17-76) | 46 | 14 | 3.8 (2-6) | - | - | - | - | - | - | - |
| Chung | 2021 | 48.7 | 105 | 21 | - | 126 | - | - | - | - | - | - |
| Consorti | 2013 | 56 | 33 | 17 | 4.1 (3.5-6) | 50 | - | - | - | 90.8 | 37 | 13 |
| Iqbal | 2014 | 45.5 | 86 | 14 | - | 10 | 38 | 52 | - | - | 100 | - |
| Jayaram | 2021 | 43.3 | 80 | 13 | - | 10 | 8 | 75 | - | - | 83 | 10 |
| Ku | 2020 | 51.8 | 30 | 7 | - | 21 | - | 16 | - | - | 4 | 33 |
| Maw | 1997 | 50.4 | 25 | 25 | 9.4 | - | - |  | - | - | - | - |
| O'Leary | 2014 | 53.5 | 69 | 13 | - | 21 | - | 23 | 36 | 102 | 74 | 8 |
| Pronio | 2011 | 42.3 | 17 | 53 | - | - | - | - |  | - | 59 | 11 |
| Rana Challa | 2020 | 48.7 (24-76) | 82 | 8 | - | 25 | 56 | 9 | - | - | 83 | 7 |
| Rao Vinay | 2021 | - | 63 | 11 | - | - | - | - |  | - | - | - |
| Reed | 1997 | 48.6 | 55 | 13 | - | - | 38 | - | 30 | - | - | - |
| Ridgway | 2009 | - | - | - | - | 5 | 6 | 14 | 4 | - | - | - |
| Selvadurai | 1997 | 51.5 | 67 | 13 | 9 | 4 | 9 | 40 | 27 | - | 80 | 0 |
| Teoh | 2019 | 52 | 71 | 25 | 6.2 | 48 |  | 17 | 31 | - | - | - |
| Vinay | 2017 | 48.6 (24-76) | 82 | 8 | - | 78 | 12 | - | - | - | 74 | 16 |
| Yang | 2013 | 44.4 | 104 | 28 | - | - | - | - | - | 55.6 | 132 | - |
|  |  | 49.2 (17-76) | 1,084 | 303 | 6.6 (2-11) | 398 | 167 | 246 | 128 | 82.3 | 726 | 98 |

TT; total thyroidectomy, STT; subtotal thyroidectomy, HT; hemi-thyroidectomy, PT; parathyroidectomy

A

B

C

D

E

F

G

H

I


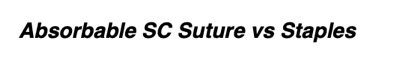

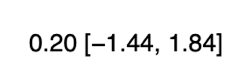

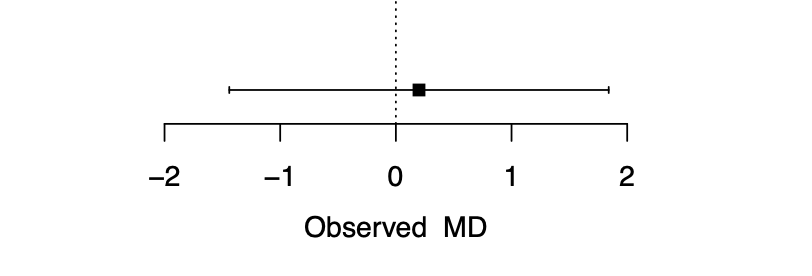


J

**Figure S1.** Study data summary and network plots of all included individual studies assessing rates of (A), overall complications, (B) infection, (C), dehiscence, (D) haematoma, (E) closure duration, (F) patient satisfaction, (G) ability to shower, (H) patient reported cosmesis, and post-operative pain expressed as (I) odds ratio and (J) mean difference.

.

| Author | Year | Cosmesis Scale | Pain Scale | Neck Mobility Scale | Risk of Bias |
| --- | --- | --- | --- | --- | --- |
| Alicandri-Ciufelli | 2014 | Wound registry scale, SBSES | - | - | Some |
| Amin | 2009 | Manchester Score | VAS | - | Low |
| Chung | 2021 | POSAS | POSAS | - | Some |
| Consorti | 2013 | POSAS | - | - | Some |
| Iqbal | 2014 | Satisfied (Yes/No) | - | - | Some |
| Jayaram | 2021 | - | - | - | Some |
| Ku | 2020 | SBSES, Manchester Score | VAS | - | Some |
| Maw | 1997 | Hollander Wound Evaluation Scale | VAS | - | Some |
| O'Leary | 2014 | Hollander Cosmesis Scale, Likert Scale | - | - | Low |
| Pronio | 2011 | SBSES | - | - | Some |
| Rana Challa | 2020 | - | VAS | - | High |
| Rao Vinay | 2021 | SBSES | VAS | - | Some |
| Reed | 1997 | - | - | - | High |
| Ridgway | 2009 | VAS | VAS | VAS | Some |
| Selvadurai | 1997 | Satisfied (Yes/No) | VAS | - | Some |
| Teoh | 2019 | POSAS, SBSES | - | - | Low |
| Vinay | 2017 | - | VAS | VAS | Some |
| Yang | 2013 | Manchester Score | VAS | - | Some |

VAS; visual/verbal analogue scale, SBSES; Stony Brook Scar Evaluation Scale, POSAS; Patient and Observer Scar Assessment Scale

**Table S2.** Included randomised clinical trials and the cosmetic, pain, neck mobility scales used and risk of bias assessment for each trial

| Author | Random Sequence Generation | Allocation Concealment | Blinding of Participants and Researchers | Blinding of Outcome | Attrition Bias | Selective Reporting | Other Biases | Risk of Bias |
| --- | --- | --- | --- | --- | --- | --- | --- | --- |
| Alicandri-Ciufelli | Low | Unclear | High | Low | Low | Low | Unclear | Some |
| Amin | Unclear | Low | Unclear | Low | Low | Low | Low | Low |
| Chung | Low | Low | High | Low | Low | Low | Some | Some |
| Consorti | Unclear | Unclear | High | Low | Low | Low | Low | Some |
| Iqbal | Unclear | Unclear | High | High | Low | Low | Low | Some |
| Jayaram | Low | Low | High | Low | Unclear | Low | Unclear | Some |
| Ku | Low | Low | Low | Low | Unclear | Unclear | Unclear | Some |
| Maw | Unclear | Unclear | High | Low | Low | Low | Unclear | Some |
| O'Leary | Low | Low | Unclear | Low | Low | Low | Low | Low |
| Pronio | Unclear | Unclear | Unclear | Low | Low | Low | Unclear | Some |
| Rana Challa | Low | Unclear | High | High | Unclear | Low | High | High |
| Rao Vinay | Unclear | Unclear | Unclear | Low | Low | Low | Unclear | Some |
| Reed | Unclear | Unclear | High | High | Unclear | Unclear | Unclear | High |
| Ridgway | Low | Low | Low | Unclear | Unclear | Low | Unclear | Some |
| Selvadurai | Low | Low | Unclear | Unclear | Low | Low | Unclear | Some |
| Teoh | Low | Low | Low | Low | Low | Low | Unclear | Low |
| Vinay | Unclear | Low | Low | Low | Unclear | Low | Unclear | Some |
| Yang | Low | Low | Low | Unclear | High | Low | Low | Some |

**Table S3.** Detailed risk of bias assessment for each included randomised clinical trial.
